# Can high social capital at the workplace buffer against stress and musculoskeletal pain? Cross-sectional study

**DOI:** 10.1097/MD.0000000000010124

**Published:** 2018-03-23

**Authors:** Kenneth Jay, Lars L. Andersen

**Affiliations:** a National Research Centre for the Working Environment, Copenhagen, Denmark; b The Carrick Institute for Graduate Studies, Institute of Clinical Neuroscience and Rehabilitation, Cape Canaveral, FL; c Sport Sciences, Department of Health Science and Technology, Aalborg University, Aalborg, Denmark.

**Keywords:** biopsychosocial, bonding, bridging, laboratory technicians, linking, pain, stress, work environment

## Abstract

Work-related musculoskeletal pain and stress are both highly prevalent in the working environment and relate well to the biopsychosocial model. While the onset of musculoskeletal pain is often dependent on the biological element of the biopsychosocial model, chronic pain is often influenced by psychological and social factors. Similarly, stress is also influenced by biological, psychological, and social factors. This study investigates the possibility of social capital being a buffer for stress and musculoskeletal pain in a group of female laboratory technicians.

Female laboratory technicians (n = 500) replied to questions about stress (Cohens Perceived Stress Scale-10), musculoskeletal pain (0–10 visual analog scale), and social capital at the workplace (bonding [in teams], bridging [between teams], and linking [between teams and leaders]). Outcome variables were stress and musculoskeletal pain and the predictor variable was social capital. General linear models tested the association of the 3 types of social capital (predictor variables) with stress and pain (mutually adjusted outcome variables). Analyses were controlled for age, lifestyle (body mass index, smoking), seniority, and working hours per week.

For stress as outcome, moderate and high bonding social capital were different from low social capital with −2.04 (95% confidence interval [CI] −3.33 to −0.76) and −4.56 (95% CI −5.84 to −3.28) points on the Perceived Stress Scale of 0 to 42, respectively. Similarly, moderate and high bridging social capital were different from low social capital with −1.50 (95% CI −2.76 to −0.24) and −4.39 (95% CI −5.75 to −3.03), respectively. For linking, only high social was significantly different from low with −2.94 (95% CI −4.28 to −1.60). None of the 3 types of social capital was associated with musculoskeletal pain.

Higher levels of social capital at the workplace appear to buffer against stress, but not against musculoskeletal pain. Intervention studies should investigate whether improving bonding, bridging, and linking social capital at the workplace may be a viable strategy to prevent or reduce work-related stress.

## 1 Introduction

The value of social networks is the central premise of social capital. For instance, Putnam defines social capital as “features of social organization such as networks, norms, and social trust that facilitate coordination and cooperation for mutual benefit”^[1]^ and Nahapiet and Ghoshal defines social capital as: “the sum of the actual and potential resources embedded within, available through, and derived from the network of relationships possessed by an individual or social unit. Social capital thus comprises both the network and the assets that may be mobilized through that network,”^[2]^ while Coleman defines it as: “Social capital is defined by its function. It is not a single entity, but a variety of different entities having two characteristics in common: They all consist of some aspect of social structure, and they facilitate certain actions of individuals who are within the structure.”^[3]^ By these definitions, social capital can therefore best be described as referring to the collective value of all “social networks” and the “norms of reciprocity” that arise from these networks. Social capital describes a variety of specific benefits that flow from the trust, reciprocity, information, and cooperation associated therewith, thus creating value and productive benefits for the people connected. By these definitions, a high social capital seems like something that should be an inherent part of a healthy environment at modern workplaces.

In relation to workplace research, 3 main types of social capital exist.^[4,5]^ The first is social capital within working teams (bonding) and represents working relationships within the team or group, for example, agreeing about what is important and a feeling of unity and cohesion in the team. The second is social capital between working teams (bridging) and represents working relationships between different teams or groups, for example, having trust in the ability of the other team to do the task well. The third is social capital between teams and leaders (linking) and represents working relationships between the members of the team or group and their leader, for example, to what degree does the leader understand and acknowledge the work of the group and whether there is a common understanding between the leader and members of the group about how to perform the work. While all 3 types are important, they represent different aspects of social capital and can respond differently to interventions at the workplace. For example, we have recently found improved bonding social capital in response to group-based physical exercise at the workplace in spite of a general decrease in linking social capital.^[4]^

Work-related stress arising from low job control and high job demands have been shown to be associated with adverse health outcomes such as hypertension and other health-risk behaviors.^[6]^ Underlying conditions of stress cross both physical/biological and psychological barriers when an organism is strained beyond its power to adapt. From a physical perspective, stress has been explained as a mechanical and automatic response from the human body and a similar response occurs when the threat has psychological characteristics.^[7,8]^ The human body has an innate drive to maintain a biological equilibrium. Stressors such as pain, sickness, or excessive physical or psychosocial work demands disrupt the homeostasis and trigger a natural response from the body, aimed at returning it to homeostasis. Briefly, the natural response can be described as a tri-phasic phenomenon. The first phase is an alarm phase representing a somatic shock followed by the second phase being a resistance to this shock where the body will fight the alarming threat and work its way back to homeostatic equilibrium. The first 2 stages are repeated throughout an individual's life as the person faces new challenges and obstacles. However, should the body remain in the second stage for prolonged periods of time, the body may enter the exhaustion stage. Models of the exhaustion stage indicate that it is the inability to adapt to the causing stressors or to an extended duration of time being subjected to the stressors that create the symptoms describing the stress state.^[7,9–12]^ The proposed model by Cooper and Marshall of work-related stress describes 5 sources of stress, each with the possibility of disrupting homeostatic equilibrium for an extended period leading to the exhaustion phase: intrinsic to the job, including factors such as poor physical working conditions, work overload, or time pressures; role in the organization, including role ambiguity and role conflict; career development, including lack of job security and under/over promotion; relationships at work, including poor relationships with the boss or colleagues, an extreme component of which is bullying in the workplace; and organizational structure and climate, including little involvement in decision-making and office politics.^[13]^ Combined, these stressors describe a biopsychosocial relationship^[14,15]^ in the development of work-related stress where perceived social interactions were social cohesion, trust, reciprocity, and cooperation in the workplace,^[1]^ affects health outcomes, such as pain,^[16]^ health-risk behaviors,^[17,18]^ depression,^[19]^ and even mortality.^[20]^ In addition to stress, musculoskeletal pain is also a major work-related challenge.

Work-related pain plays a dominating role in work environment and health.^[21,22]^ It is a major socioeconomic burden with consequences for both the individual, the social relations of the individual, and the organization.^[23,24]^ Musculoskeletal pain is one of the most common causes for loss of productivity, reduced work performance, and sickness absence.^[25]^ In addition, chronic pain has been associated with poor quality of life.^[26–28]^ Among laboratory technicians, the prevalence of chronic musculoskeletal pain is high.^[29–31]^ This type of job is characterized by tasks that are monotonous, with relatively low force, continuous muscular contractions, and repetitive in nature. Large epidemiological studies following several thousand workers in registers have shown that repetitive arm movements for more than 25% of the working time is a risk factor for developing long-term sickness absence.^[32]^

The biopsychosocial model of pain explains the intricacies and interplay of biological/biomedical, psychological, and social factors affecting pain^[14,15]^ and while there is some evidence that show biological factors are predominant at the onset of pain, psychological and social factors are central in the process of developing pain chronicity.^[33,34]^ For instance, social support,^[35]^ which can be regarded as a part of social capital, has been shown to be helpful in chronic pain coping,^[36,37]^ whereas lack of social support augments the development of chronicity.^[38]^ With the biopsychosocial model of both stress and pain in work, environment, and health, it therefore seems relevant to ask the question; can high social capital at the worksite buffer against stress and musculoskeletal pain in a population performing monotonous and repetitive movement tasks such as laboratory technicians?

## 2 Methods

### 2.1 Study design

This study is an explorative cross-sectional analysis of baseline data obtained during a worksite intervention trial previously described by our research team.^[29–31,39,40]^ Data for this study were collected during the spring of 2014. A protocol of the study and primary, secondary, and tertiary outcomes, together with a cross-sectional study of work ability, have all been reported previously.^[29–31,39,40]^

### 2.2 Ethical approval

Ethical approval was obtained from the Danish National Committee on Biomedical Research Ethics (the local ethical committee of Frederiksberg and Copenhagen; H-3-2010-062) as part of the research program “Implementation of physical exercise at the workplace (IRMA).” The trial “Implementation of physical exercise at the Workplace (IRMA09)—Laboratory technicians” was registered in the ClinicalTrials.gov register (NCT02047669) prior to participant enrolment. All experimental conditions conformed to the Declaration of Helsinki. All reporting conforms to the STROBE guidelines “Strengthening the Reporting of Observational Studies in Epidemiology.”^[41]^

### 2.3 Participants

Out of 756 laboratory technicians at a large pharmaceutical company in Denmark, 539 completed questionnaires on musculoskeletal pain, perceived level of stress, and social capital. Of these, 473 were women and included in the analysis. Table 1 shows participant demographics of relevant data. All eligible participants were informed about the purpose and content of the study. Table 1 shows participant characteristics of relevant data.

**Table 1:**
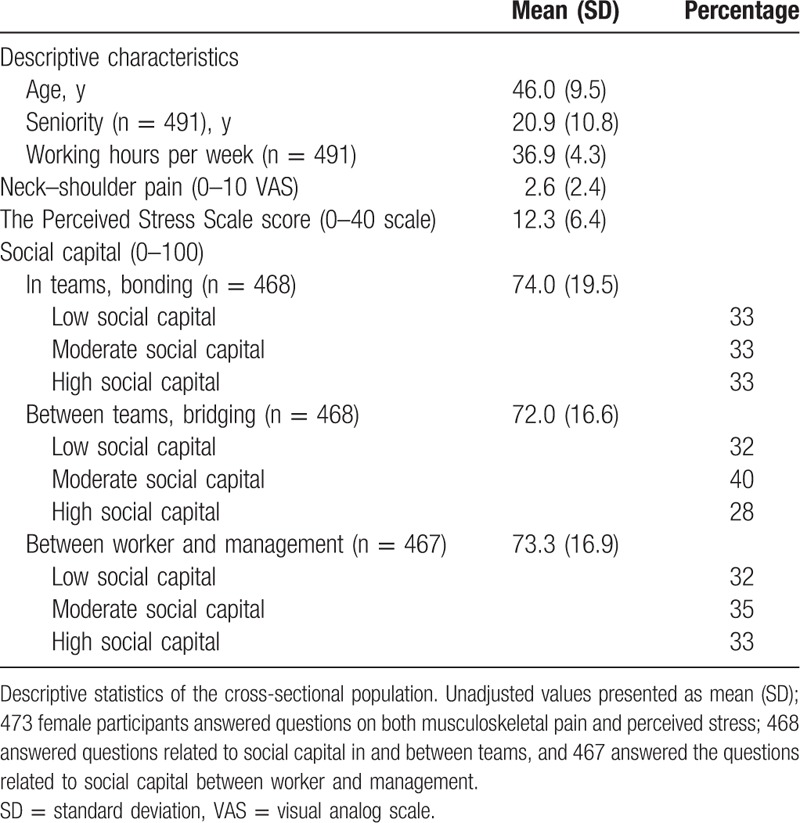
Descriptive characteristics.

### 2.4 Outcome variables

#### 2.4.1 Stress

The Perceived Stress Scale (PSS) is a comprehensive stress questionnaire and was designed to measure “the degree to which individuals appraise situations in their lives as stressful.” Items evaluate the degree to which people find that life is unpredictable, uncontrollable, or overloaded.^[42]^ These 3 aspects have been confirmed as vital elements of the experience of stress and provide a thorough insight into the degree of learned helplessness experienced by the individual.^[43]^ The Perceived Stress Scale 10 (PSS-10) includes questions intended to evaluate the current level of stress experienced by the subject and is an abbreviated version of the scale, consisting of only 10 items (the full version has 14 items), administered in only a few minutes, and easily scored. Because the PSS assesses general beliefs about perceived stress without providing subjects with a list of specific life events, scores are not biased by event content or by differential recall of previous life experiences. In brief, each item on the PSS-10 questionnaire is rated on a 5-point Likert scale ranging from “never” (0) to “almost always” (4). Positively worded items are reverse scored, and the ratings are summed, with higher scores indicating more perceived stress. The PSS-10 score is obtained by reversing the scores on the 4 positive items: For example, 0 = 4, 1 = 3, 2 = 2, etc. and then summing across all 10 items. A score of 13 is considered average and stress scores of more than 20 indicate high stress.^[29]^ For reference, we divided the scoring into 3 categories with the following cut-off points: low stress ≤ 10, 10 < moderate stress ≤ 20, and high stress > 20. Examples of questions from the PSS-10 questionnaire include: In the past month, how often have you been angry because of things that happened that were outside of your control?, In the past month, how often have you felt that things were going your way?,” and “In the past month, how often have you felt unable to control the important things in your life?”^[42]^

#### 2.4.2 Musculoskeletal Pain

We asked the participants to rate their pain intensity in the upper back, lower back, neck, shoulders, elbows, or hands/wrists on a modified 0 to 10 visual analog scale.^[44]^ For reference, “0” is defined as “no pain” and “10” is defined as “worst imaginable pain.” The questions were supported by drawings from the Nordic Questionnaire that defined the body areas^[45]^ and an average pain score of the 6 regions was subsequently calculated and used in the analysis.

### 2.5 Predictor variables: Bonding, bridging, and linking

Female workers (n = 473) replied to a baseline screening questionnaire concerning Bonding, Bridging, and Linking (A + B) social capital.^[4,5]^ Two sample questions out of 9 questions for bonding social capital are “In our team, we agree on what is the most important in our work tasks” and “There is a feeling of unity and cohesion in my team.” Two sample questions of a total of 6 for bridging social capital are “Is there a good working relationship between your team and the other teams/departments?” and “We have trust in the ability of the other teams to do the job well.” Two sample questions out of 10 questions for linking social capital are “Do your nearest leader contributes to solving everyday problems?,” “Our nearest leader has great knowledge and understanding of the work we do,” “Are the employees involved in decisions about changes at the workplace?,” and “There is a common understanding between the management and employees on how we should perform our work tasks.” Participants replied on a horizontally oriented scale of 0 to 10, where 0 is “no, not at all” and 10 is “Yes, completely.” For each of the social capital dimensions, the average value of all questions was calculated and multiplied by 10 (i.e., 0–100) to provide a higher resolution of the respective social capital dimension.^[4]^ The cut points between low, moderate, and high social capital in bonding, bridging, and linking were chosen to have as close to 33.3% of the subjects in each group—in teams, low social capital (0–69), moderate social capital (69–85), and high social capital (85–100); between teams: low social capital (0–66), moderate social capital (66–80), and high social capital (80–100); and between teams and leader/management: low social capital (0–69), moderate social capital (69–82), and high social capital (82–100).

### 2.6 Statistics

General linear models (Proc GLM, SAS version 9.4) tested the association of the 3 types of social capital (predictor variables) with stress and pain (mutually adjusted outcome variables). Analyses were controlled for age, lifestyle (body mass index [BMI], smoking), seniority, and working hours per week. Stress analysis was controlled for pain, and pain analysis was similarly controlled for stress. Results are reported as least square means and 95% confidence intervals (CIs), as well as between-group differences of least square means and 95% CIs. In addition, effect sizes (Cohen d) were calculated as the between-group difference (high vs. low, and moderate vs. low) divided by the pooled standard deviation.^[46]^ According to Cohen, effect sizes of 0.20, 0.50, and 0.80 can be considered small, moderate, and large, respectively.

## 3 Results

For stress as outcome, moderate and high social capitals in teams are both statistically different from low social capital with effect sizes of 0.32 and 0.71, respectively. A similar picture is seen with social capital between teams where moderate and high social capitals are statistically different from low social capital with effect sizes of 0.23 and 0.69, respectively. For social capital between team and leader/management, only high social capital buffers against stress compared with low social capital with an effect size of 0.46. With musculoskeletal pain as outcome neither moderate or high social capital in bonding, bridging, or linking buffers against musculoskeletal pain compared with low social capital. Full results on social capital as buffer against stress and pain are shown in Tables 2 and 3, respectively. Thus, social capital appears to buffer against stress but not musculoskeletal pain.

**Table 2:**
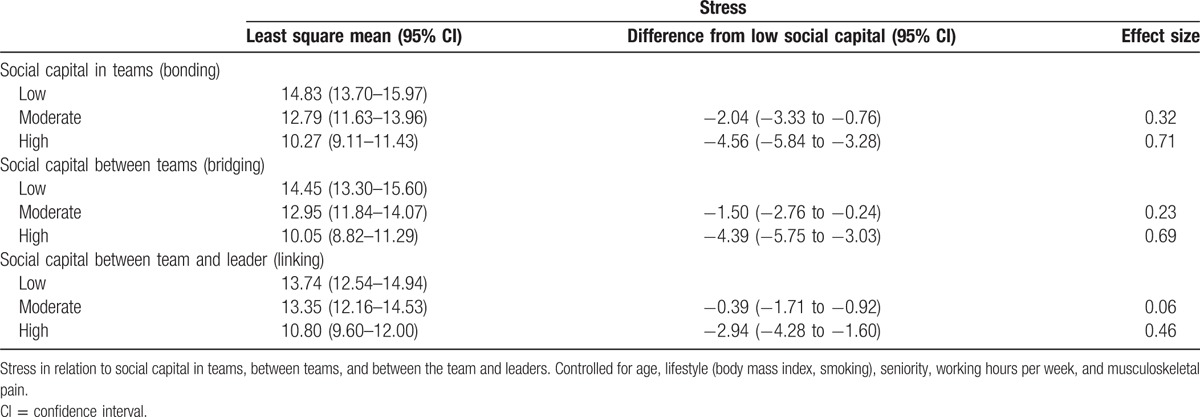
Stress and social capital.

**Table 3:**
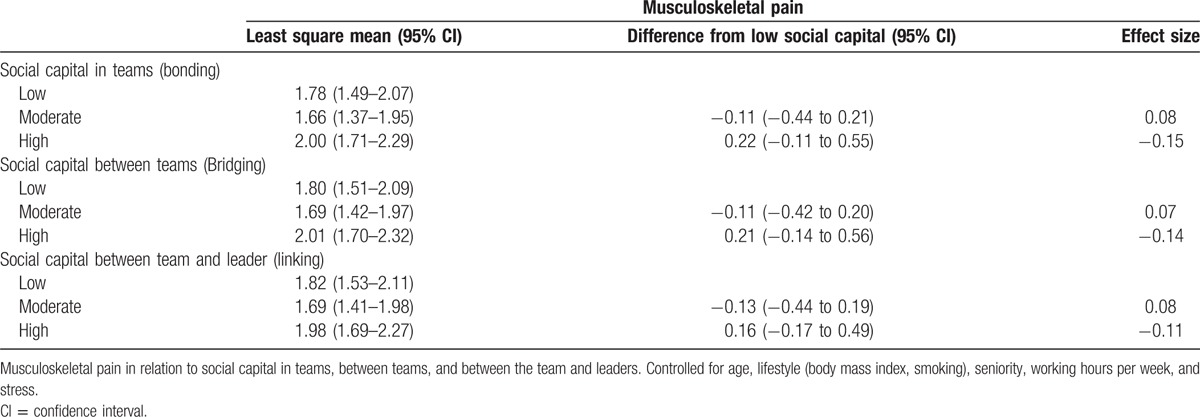
Pain and social capital.

## 4 Discussion

The present cross-sectional analysis of approximately 500 female laboratory technicians shows that having both moderate and high social capital buffers against perceived stress, overall with low to moderate effect sizes, but not against work-related musculoskeletal pain when adjusting for age, lifestyle (BMI, smoking), seniority, and working hours per week. This finding has significant implications for testing future targeted worksite rehabilitation strategies focusing on both work-related chronic musculoskeletal pain and stress. Our research team has previously shown that a multifactorial intervention strategy targeting musculoskeletal pain and stress by utilizing precise joint mobility and elastic resistance band exercises, fear-avoidance and pain catastrophizing counseling, and mindfulness at the worksite reduces chronic musculoskeletal pain by 52%, and reduces fear avoidance beliefs by 23% but does not significantly reduce perceived levels of situational stress, neurocognitive performance or muscle function compared with a reference group following on-going company health initiatives. Further, we have also shown that work ability in the same population is affected by both stress and pain in an additive fashion.

This study provides an interesting perspective on the relationship between social capital and chronic pain. For instance, low cognitive social capital at individual level has been shown to be significantly associated with a higher prevalence of pain and higher level of pain intensity, but also with a higher chance for sick leave due to pain in employed subjects.^[16]^ In addition, others have shown that increasing the social capital in cancer patients results in promoting health behaviors, treatment compliance, and pain relief^[47]^ and recently it was shown that physical exercise programs performed together with colleagues improve social climate and vitality among workers with chronic musculoskeletal pain but did not affect mental health,^[48]^ which suggests that focusing on improving social capital can be beneficial in persons with chronic pain, this study suggests that having moderate or high social capital does not act as a buffer against musculoskeletal pain. Therefore, increasing social capital as a preventive measure of work-related musculoskeletal pain development alone may not be a feasible strategy but should still be considered for rehabilitation purposes. Conversely, this study does suggest that having moderate or high social capital does buffer against stress. This perspective is interesting as it implies an important aspect of work health and it supports both the psychological and social elements of the biopsychosocial model as described by Engel in 1977^[14,15]^ and since stress can be described as either the reaction (psychological, physiological, and behavioral) to environmental stimuli or the interaction between environmental characteristics and the subjective reaction to these characteristics,^[31,49]^ improving social capital at the worksite appears to be a viable strategy to implement when the goal is to buffer against work stress. This is supported by the findings of Boyas and Wind who are, in a cross-sectional study, examined the relationship between employment-based social capital, job stress, and burnout among public child welfare workers and found that communication, supervisory support, organizational commitment, influence, and trust—all elements of social capital—had a significant association with job stress.^[50]^ Furthermore, Gächter et al found, by analyzing survey data from police officers to assess the relationship between stress, strain, and social capital, that an increase in social capital is significantly correlated to a decrease in perceived level of strain and psychological burnout, thus recommending that stress reduction programs should actively engage employees to build stronger social relations and networks.^[51]^

Although all 3 types of social capitals buffered against perceived stress, social capital in and between teams appeared to be more important than social capital between leaders and teams, that is, both moderate and high levels of social capital were important. Thus, factors such as agreeing on what is the most important in daily work tasks and a feeling of unity and cohesion in the team seem to be important within the teams, while good working relationships and trusting other teams seem to be important between teams. By contrast, only high social capital, and not moderate social capital, between teams and leaders were important for lower levels of perceived stress. Thus, a really high level of contribution from the leader to solve everyday problems, a great knowledge and understanding of the work that the employees do, involving employees in decisions about changes at the workplace, and having a common understanding about what is expected are important factors between leaders and teams in relation to lower levels of stress.

### 4.1 Strengths and limitations

This study demonstrates that moderate and high social capital buffers against work stress but not against musculoskeletal pain. However, the study has some important limitations. The cross-sectional design does not allow examination of causal associations. Although the sample size is adequate to test the research question, self-reported data are a limitation as they may be influenced by subjective factors. Finally, given the demographic characteristics of this sample (Danish female laboratory technicians), generalizability to other job groups, to men, and to other countries remain to be determined. Contrariwise, using a homogenous sample consisting of female laboratory technicians is also a noteworthy strength, as it limits bias from socioeconomic confounding. Finally, because social capital deals with human behavior, the study could have been strengthened by qualitative interviews to supplement the questionnaire replies.

In conclusion, this study provides an interesting perspective in factors that may be important in stress prevention and management at the worksite. Our results indicate that having moderate and high social capital buffer against stress. Intervention studies should test whether improving social capital in bonding, bridging, and linking may be a viable strategy to implement in a time where work-related stress is highly prevalent and a socioeconomic burden of considerable size.

## 5 Author contributions

**Conceptualization:** K. Jay, L.L. Andersen.

**Data curation:** K. Jay, L.L. Andersen

**Project administration:** K. Jay.

**Writing – original draft:** K. Jay.

**Writing – review & editing:** K. Jay, L.L. Andersen.

**Formal analysis:** L.L. Andersen.

**Supervision:** L.L. Andersen.
